# Diagnostic evaluation of risk for bleeding in cardiac surgery with extracorporeal circulation*

**DOI:** 10.1590/1518-8345.2523.3092

**Published:** 2018-11-29

**Authors:** Damaris Vieira Braga, Marcos Antônio Gomes Brandão

**Affiliations:** 1 Universidade Federal do Rio de Janeiro, Escola de Enfermagem Anna Nery, Rio de Janeiro, RJ, Brazil.

**Keywords:** Thoracic Surgery, Extracorporeal Circulation, Risk Factors, Nursing Diagnosis, Hemorrhage, Postoperative Care

## Abstract

**Objective:**

to identify the risk factors associated with cases of excessive bleeding in patients submitted to cardiac surgery with extracorporeal circulation.

**Method:**

case-control study on the factors of risk for bleeding based on the analysis of data from the medical charts of 216 patients submitted to cardiac surgery with elective extracorporeal circulation during a three-year period.

**Results:**

variables that are commonly associated with excessive bleeding in studies in the field were analyzed, and the following were considered as risk factors for the nursing diagnosis “risk for bleeding” (00206) in cardiac surgery with extracorporeal circulation: Body mass index lower than 26.35kg/m² (Odds ratio = 3.64); Extracorporeal circulation longer than 90 minutes (Odds ratio = 3.57); Hypothermia lower than 32°C (Odds ratio = 2.86); Metabolic acidosis (Odds ratio = 3.50) and Activated partial thromboplastin time longer than 40 seconds (Odds ratio= 2.55).

**Conclusion:**

such variables may be clinical indicators of an operational nature for a better characterization of the risk factor “treatment regimen” and a refinement of knowledge related to coagulopathy induced by extracorporeal circulation, which is currently presumably incorporated into the “treatment regimen” category of the nursing diagnostic classification by NANDA International, Inc.

## Introduction

Bleeding is a common and severe occurrence in the postoperative period of cardiac surgeries. Excessive losses tend to require corrective measures, such as transfusions, thus leading to complications, as for instance: surgical reexploration, increased mortality after 30 days, and prolongation of mechanical ventilation for more than 24 hours^1^ ^-^ ^3^. Hence, in recent years, there has been investment in the creation and use of more appropriate protocols to improve hemostasis^4^. The literature has presented bleeding risk factors or elements that indicate the need for transfusion in cardiac surgery, among them: the use and the discontinuation time of the use of antithrombotic agents before surgery, replacements with colloidal solutions, techniques and equipment for extracorporeal circulation (ECC), comorbidities, clinical conditions, such as coagulopathies and hemoglobinopathies and chronic diseases, such as high blood pressure, renal failure and diabetes, among other factors^4^ ^-^ ^6^.

Despite the advances made by studies on the factors of risk for bleeding in adults undergoing cardiac surgery with ECC, there are still several areas of uncertainty and topics for additional studies^7^, which can be partly explained by the patients’ preoperative clinical conditions and the multiplicity of therapeutic interventions that demarcate the perioperative situation, as well as the complexity involved in cardiac surgeries and the difficulty in studying their variables separately.

The relevance of bleeding as a professional focus of nursing is recognized in the NANDA International, Inc. taxonomy by the incorporation of the nursing diagnosis (00206) “risk for bleeding” defined as the “vulnerability to reduction in blood volume that may compromise an individual’s health”^8^. Among the factors of risk for bleeding presented in the nursing diagnosis is the inclusion of the generic concept of “treatment regimen”, which could embrace elements related to cardiac surgery conditions. However, for the nurse operating in the perioperative period of cardiac surgery, the concept of treatment regimen requires detailed information on what would be the significant operational elements related to bleeding in the postoperative period of cardiac surgeries.

A study published by nurse researchers demonstrated the factors associated with excessive bleeding after cardiac surgery, thus identifying significant factors related to excessive bleeding and advancing the knowledge on the subject. The authors, however, did not explicitly correlate the results to the nursing diagnosis risk for bleeding^9^. Nevertheless, despite the evidence already obtained, some variables have different values of relevance in the estimation of risk for bleeding, and different criteria are adopted for the definition of excessive bleeding^2^ ^,^ ^6^ ^,^ ^10^ ^-^ ^12^. Therefore, it is understood that further studies are required in order to base an accurate diagnosis by nurses so as to provide valid operational criteria.

In view of the above, this article aims to identify the risk factors associated with cases of excessive bleeding in patients submitted cardiac surgery with ECC.

## Method

This is a case-control study on the factors of risk for bleeding conducted at a general federal tertiary military hospital located in the city of Rio de Janeiro, southeastern Brazil. Patients undergoing elective cardiac surgery with ECC were investigated. The study was approved by the Research Ethics Committee under registration number 55217516.2.0000.5238.

Variables potentially associated with postoperative bleeding were selected from the taxonomy of NANDA International, Inc.^8^ and from a literature review. Variables mainly found in studies whose characteristics converged to those of the clientele and institution investigated were preferentially selected^3^ ^-^ ^6^ ^,^ ^10^.

**Figure 1:**
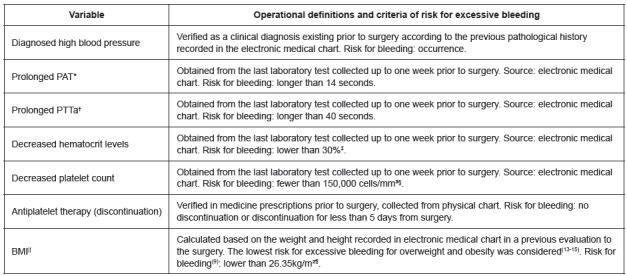
Preoperative variables selected to be tested as risk factors for bleeding. Rio de Janeiro, RJ, Brazil, 2013-2015

The preoperative variables selected for the study as risk factors are presented in Figure 1.

*Prothrombin activation time; †Activated partial thromboplastin time; ‡Percentage; §Cubic millimeters; ||Body mass index; ¶Kilos per square meter

**Figure 2:**
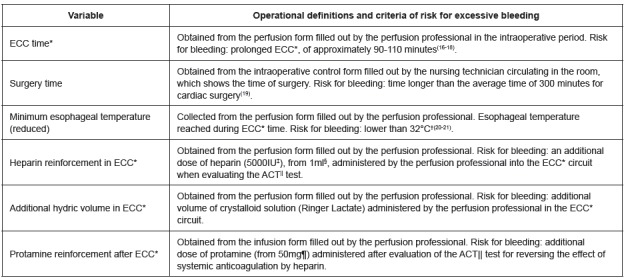
Intraoperative variables selected to be tested as risk factors for bleeding. Rio de Janeiro, RJ, Brazil, 2013-2015

The intraoperative variables selected for the study as risk factors are presented in Figure 2.

*Extracorporeal circulation; †Degrees Celsius; ‡International units; §Milliliters, ||Activated coagulation time; ¶Milligrams

**Figure 3:**
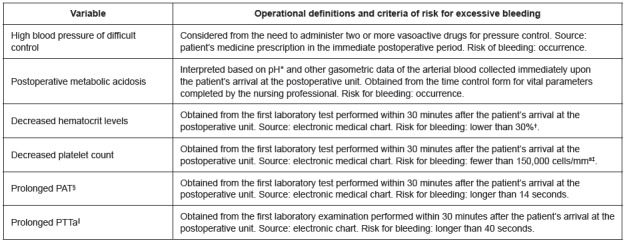
Postoperative variables selected to be tested as risk factors for bleeding. Rio de Janeiro, RJ, Brazil, 2013-2015

The postoperative variables selected for the study as risk factors are presented in Figure 3.

*Hydrogen potential; †Percentage; ‡Cubic millimeters; §Prothrombin activation time; ||Activated partial thromboplastin time

Studies and clinical practice have adopted different definitions and criteria to define abnormal perioperative bleeding, such as drainage through the thoracic tubes, magnitude of blood transfusions, delayed sternal closure, and use of coagulation concentrates^2^. The present study adopted the concept of excessive bleeding as a function of the volume drained by the thoracic tubes because it is aligned with a nursing intervention and more easily articulated to the nursing diagnosis. The following were adopted as criteria for excessive bleeding: bleeding from mediastinal and/or pleural tubes with values equal to or greater than 1.5ml drainage/kg/h for at least 3 hours^11^, or drainage greater than 200ml/h or fraction of an hour or greater than 2ml/kg/hour for two consecutive hours in the first 6 hours postoperatively^3^. Blood loss was accompanied by hourly measurements of mediastinal and/or pleural drainage during the first 24 hours, with values verified and documented by the nursing team.

Data were collected by one of the researchers throughout 2016 from the medical charts of all individuals undergoing cardiac surgeries at the institution between 2013 and 2015. The time interval is due to the full implantation of electronic medical charts in the institution in order to facilitate information retrieval, the quality and truthfulness of the data collected.

The collected data were recorded in a collection instrument created with the purpose of characterizing the profile of the population submitted to cardiac surgery at the said hospital, in addition to characterizing the variables that occur at the different perioperative moments of cardiac surgery. To that end, this instrument contained information on the population’s profile as well as their clinical, surgical and postoperative data. The possibility of occurrence of information bias due to errors in recording data in the medical charts was considered. However, it should be noted that some conditions of data production probably minimized such risk, among them: the qualification and technical preparation of the team responsible for the documentation and the exclusive allocation of a nurse and a physician for the care provided during the immediate postoperative phase, including record making and the use of a standardized device for accurate blood drainage measurement.

The medical charts of patients who had undergone elective cardiac surgery with ECC available for consultation at *Seção Arquivo Médico* ^1^ (SAM) of the institution during the data collection period were eligible. Medical charts with incomplete records were excluded from the study.

Of a total of 275 cardiac surgeries performed between 2013 and 2015, 269 were elective with ECC, thus constituting the potentially eligible sample. Fourteen medical charts with incomplete information and 22 that were not available for consultation were discarded. There were three death losses during the perioperative period; therefore, 230 charts remained.

Based on the excessive-bleeding criteria, the occurrence of 24 cases was observed, and the case/control ratio was established as 1 case to 8 controls, so as to maximize the statistical power of the study in view of the available sample^22^ ^-^ ^23^. One hundred and ninety-two medical charts were drawn as control.

The chi-square tests or Fisher’s test were used to test the differences between the proportions obtained for the groups of cases and controls. In order to evaluate the risk of the variables for bleeding, the odds ratios (OR) and the 95% confidence intervals were calculated. The variables that obtained an odds ratio above 1.0 with *p* < 0.05 were considered as risk factors with statistical significance. The data were analyzed by the MedCalceasy-to-use statistical software®.

## Results

The 216 participants were mostly elderly men, which is in agreement with the characteristics observed in similar studies^1^ ^,^ ^3^ ^,^ ^5^ ^,^ ^9^ ^,^ ^12^ ^-^ ^13^ ^,^ ^18^. Regarding the surgery performed, there was a predominance of revascularization surgeries, with 50% among cases and 70.83% among controls, followed by valve replacement (29.17% for cases and 27% for controls), combined surgeries (20.83% for cases and 14.06% for controls), atrial septal defect correction, myxoma resection and Bentall-De Bono surgery, thus comprising the remaining 3.13% of controls. The comorbidities observed were: diabetes mellitus (37.5% of cases and 62% of controls), chronic renal failure (4.2% of cases and 3.5% of controls) and coagulopathies in 1.04% and hemoglobinopathies in 0.52% of controls. None of the characterization data contributed to the chance of excessive bleeding in the sample.

Table 1 shows the occurrence data for the excessive-bleeding risk variables as a function of the condition, such as case-group and control-group.

**Table 1:** Variables associated with the group with excessive bleeding (cases) and without excessive bleeding (controls). Rio de Janeiro, RJ, Brazil, 2013-2015

Variables	Cases		Controls		*p* value*
	n = 24		n = 192		
Categorical	n	%	N	%	*p* ^†^
Male	18	64.60	135	69.40	0.8125
Female	6	35.40	57	30.60	0.6344
Systemic high blood pressure	20	83.30	170	88.50	0.4627
Additional hydric volume in ECC^‡^	14	58.30	104	54.10	0.6975
Return to the operating room	7	29.17	0	0	< 0.0001^§^
Post-ECC^‡^ platelet transfusion	2	8.33	20	10.9	0.7513
Numerical	mean (standard deviation)		mean (standard deviation)		*p* ^||^
Preoperative period					
Age (years)	66.88 (8.64)		65.89 (10.84)		0.5316
Weight (kg^¶^)	66.66 (9.05)		75.21 (14.33)		0.0005^§^
Body mass index (kg/m^2^**)	24.35 (2.54)		27.21 (4.49)		0.0031^†^
Hematocrit (%^††^)	41.25 (4.44)		39.19 (5.42)		0.0663
Platelet count (cell/mm^3‡‡^)	212125 (60.57)		226890 (65.50)		0.1924
Hemoglobin (mg/dl^§§^)	13.98 (1.46)		13.19 (1.98)		0.0370^§^
Prothrombin activation time (seconds)	13.57 (0.65)		14.05 (3.28)		0.7247
Activated partial thromboplastin time	38.38 (5.72)		38.84 (9.68)		0.3782
Intraoperative period					
Reduced minimum esophageal temperature (°C^||||^)	30.57 (2.90)		32.06 (1.37)		0.0121^§^
Heparin reinforcement (IU^¶¶^)	5291.71 (62.82)		2591.15 (43.82)		0.0066^†^
Protamine reinforcement (mg***)	18.75 (35.55)		28.13 (39.90)		0.2730
Surgery time (minutes)	304.88 (75.74)		318.75 (96.12)		0.2891
ECC^‡^ time (minutes)	112.25 (31.45)		99.14 (31.53)		0.1296
Postoperative period					
Hematocrit (%^††^)	34.26 (4.40)		35.43 (4.33)		0.2135
Platelets (cells/mm^3‡‡^)	165.160 (52.18)		179.080 (53.96)		0.2333
Hemoglobin (mg/dl^§§^)	11.75 (1.62)		11.99 (1.52)		0.9626
Prothrombin activation time (seconds)	16.11 (1.40)		15.89 (1.92)		0.5774
Activated partial thromboplastin time	41.87 (7.33)		38.03 (6.35)		0.0019^§^

*p value; †Significance test referring to Fisher’s Test; ‡Extracorporeal circulation; ^§^Statistical significance; ||Significance test referring to the Paired t-test; ¶Kilos; **Kilos per square meter; ††Percentage; ‡‡Cubic millimeters; §§Milligrams per deciliter; ||||Celsius; ¶¶International units; ***Milligrams

Among the categorical variables, “return to the operating room” showed a higher proportion of occurrences in the case-group as compared to the control-group with statistical significance. Regarding the numerical variables, the differences between the means with statistical significance, indicating a greater association with risk for excessive bleeding in the case-group were: weight, Bodly Mass Index (BMI), reduced minimum esophageal temperature and heparin reinforcement, with values indicating risk for the case-group. Preoperative hemoglobin showed a lower mean in the control-group, together with statistical significance.

Table 2 shows the odds ratio for the variables to represent a risk factor for excessive bleeding in the postoperative period of cardiac surgery with ECC. For this purpose, the absolute and percent frequency values, odds ratio, confidence interval and *p*-value of the analyzed variables are indicated.

**Table 2:** Analysis of the association between the risk factor and bleeding, expressed by the Odds ratio. Rio de Janeiro, RJ, Brazil, 2013-2015

Risk factors	Cases	Controls	Odds ratio and significance		
	N	n	OR*	CI^†^ 95%	*p* value^‡^
Preoperative period					
Diagnosed systemic high blood pressure	20	170	0.64	(0.20 to 2.06)	0.4627
Platelet antiaggregation < 5 days	22	62	0.90	(0.35 to 2.29)	0.8353
Prothrombin activation time > 14 seconds	16	38	0.75	(0.28 to 1.98)	0.5650
Activated partial thromboplastin time > 40 seconds	13	38	1.24	(0.50 to 3.07)	0.6343
Hematocrit > 30%^§^	0	10	0.35	(0.02 to 6.24)	0.4788
Platelets < 150,000 cells/mm^3||^	6	18	1.72	(0.53 to 5.53)	0.3633
Body mass index < 26.35kg/m²^¶^	19	98	3.64	(1.31 to 10.15)	0.0134**
Intraoperative period					
ECC^††^ > 90 minutes	46	121	3.57	(1.17 to 10.85)	0.0247**
Surgery > 300 minutes	27	72	1.15	(0.49 to 2.71)	0.7364
Esophageal temperature < 32°C^‡‡^	34	55	2.86	(1.16 to 7.00)	0.0215**
Additional hydric volume in ECC^††^	29	78	1.18	(0.50 to 2.79)	0.6993
Heparin reinforcement in ECC^††^	25	54	2.28	(0.96 to 5.40)	0.0607
Postoperative period					
High blood pressure of difficult control	17	45	0.73	(0.23 to 2.27)	0.5951
Metabolic acidosis	18	58	3.50	(1.35 to 9.05)	0.0098**
Hematocrit < 30%^§^	8	14	2.39	(0.80 to 7.14)	0.1172
Platelets < 150,000 cells/mm^3||^	19	42	1.90	(0.80 to 4.50)	0.1409
Activated partial thromboplastin time > 40 seconds	21	39	2.55	(1.08 to 6.03)	0.0324**
Prothrombin activation time > 14 seconds	44	124	1.57	(0.34 to 7.10)	0.5573

*Odds ratio*;* †95% confidence interval; ‡*p* value; §Percentage; ||Cubic millimeters; ¶Kilos per square meter; **Statistical significance; ††Extracorporeal circulation; ‡‡Degrees Celsius

The variables that, due to their increased odds ratio, can be considered as risk factors in the preoperative period are: BMI lower than 26.35kg/m² (OR = 3.64); in the intraoperative period: ECC longer than 90 minutes (OR = 3.57); Esophageal temperature lower than 32°C (OR = 2.86); in the postoperative period: Metabolic acidosis (OR = 3.50); Activated partial thromboplastin time longer than 40 seconds (OR = 2.55).

## Discussion

The study found variables that were associated with excessive bleeding after cardiac surgery with ECC in the preoperative and intraoperative periods and in the first postoperative minutes. BMI lower than 26.35kg/m^2^, ECC time longer than 90 minutes, esophageal temperature lower than 32°C, and metabolic acidosis and activated partial thromboplastin time longer than 40s were validated factors and that have already been identified in other studies^2^ ^-^ ^4^ ^,^ ^6^ ^,^ ^9^ ^,^ ^12^ ^,^ ^24^. On the other hand, there were variables that did not reach statistical significance values in the present study, but were considered to be associated with excessive bleeding in the abovementioned investigations.

The lack of agreement among the findings about the factors of risk for bleeding points to the importance of research on the subject as much as it raises questions about the standardization of criteria linked to the risk for bleeding in patients undergoing cardiac surgeries. The standardization of defining criteria for abnormal or excessive bleeding is one of the aspects that points to a careful interpretation of the data in studies on excessive bleeding. For example, the criteria used in studies include various operational strategies, such as monitoring drainage through thoracic tubes using standard values in ml/h or ml/kg/h or associated with other indicators, such as follow-up on delayed sternal closure and evaluation of the use of transfusions^2^ ^-^ ^3^ ^,^ ^9^.

The results obtained in the present study were encouraging in relation to the criterion adopted to define excessive bleeding, especially because it was observed that all the patients who needed surgical reexploration were in the case-group. In a way, this shows a practical value of the criterion selected to establish the phenomenon of excessive bleeding.

Regarding BMI values, the studies present different cut-off points to establish the value that defines the excessive-bleeding condition in the postoperative period or with hemorrhagic complications, such as for example: lower than 20kg/m^2(^ ^24^, lower than or equal to 24kg/m^2(^ ^15^, 25±3kg/m^2(^ ^12^ and lower than 26.35kg/m^2(^ ^9^. We chose to use the value lower than 26.35 kg/ m^2^ as a characterization of the risk factor when considering the means for the case- and control-groups. Once the variable in question was tested, an increased odds ratio was obtained with statistical significance, which was essential to consider it a diagnostic risk factor. Despite the differences among the BMI values that would be related to excessive bleeding, there is something in common among the studies, namely: the predictive character that low weight increases the risk for bleeding. Using the results of the abovementioned studies, an assumption is made that patients submitted to ECC are more susceptible to the effects of changing coagulation factors during hemodilution^6^. Consideration should be given to the possible risks of associating low BMI with high crystalloid infusion, as an example, of the implications for the professionals involved, including perfusion professionals.

The relationships in the increased postoperative bleeding attributed to risk factors of low esophageal temperature, metabolic acidosis and changes in activated partial thromboplastin time are undesired consequences of extracorporeal circulation^6^. ECC produces a set of responses related to the interaction involving inflammatory reactions, fibrinolysis and coagulation, and hemodilution and increased consumption of coagulation factors due to increased fibrinolysis may be the cause of ECC-induced coagulopathy^25^.

Metabolic acidosis and hypothermia induced by ECC contribute to exacerbate changes in the coagulation chain^25^. Such alterations associated with the other risk factors probably close a multifactor mechanism that culminates in the increased mean of activated partial thromboplastin time (PTTa), which was observed in the postoperative period of patients in the case-group. PTTa is one of the laboratory tests that comprise the evaluation standard for management of post-ECC coagulopathy^26^ and, in light of the results, it becomes a clinical risk-factor indicator to be evaluated for the definition of the nursing diagnosis risk for bleeding in the postoperative period.

The heparin reinforcement administered, on average, at a larger dose to the case-group when compared to the control-group (Table 1) may be related to excessive bleeding. This hypothesis gains strength when we consider that a longer ECC time also increases the need for heparin administration (heparin reinforcement)^6^.

It is understood that the study brings relevant contributions to the refinement of nursing diagnosis risk for bleeding, as it allows for the application of the concept in the field of cardiac surgery by providing operational elements for the best use of risk factor “treatment regimen” and clinical adequacy of ECC-induced coagulopathy as a substitute for risk factor “inherent coagulopathy”^8^. Coagulopathy is due to coagulation disorders that include complications related to trauma or are inherent to the patient, such as thrombocytopenia, for example^8^ ^,^ ^27^, and it cannot be considered to be in the same class as the coagulopathies related to extracorporeal circulation. Additionally, there is, according to the classification by the Diagnosis Development Committee (DDC), the potential contribution from the identification of risk factors by means of clinical studies (validation and testing), level 3 of evidence^8^.

For the nursing diagnostic evaluation, the study supports the relevance of measurement or monitoring actions that can be performed by the nursing team, which contributes to increase the nurse’s diagnostic accuracy in the early detection of risk for bleeding in the immediate postoperative period as well as to promote the perception of the importance of the nursing diagnosis in collaborating with useful information to the practice of physicians and other members of the health care team. For nurses working in cardiology units, the study provides clinical information that can base a better selection of nursing interventions and more effective decision making in the monitoring of excessive-bleeding signs.

The corroboration of the findings in the present study by other investigations conducted by nurses and physicians tend to broaden the interest potential of the topic to action based on possibilities of multidisciplinary research collaboration, with gains for the advancement of knowledge in the area.

It is understood that the main limitation of the study was related to its retrospective nature, especially regarding the potential bias of information inaccuracy. However, the low occurrence of bleeding at the institution chosen for the study was one of the criteria that motivated the choice of the case-control research design. The authors assume that the data production conditions, already presented in the method section, may have minimized such methodological limit, which is difficult to overcome in the type of study developed.

## Conclusion

Considering the findings in the present study, it was concluded that the variables associated with excessive bleeding after cardiac surgery with extracorporeal circulation were: BMI lower than 26.35kg/m^2^, ECC time longer than 90 minutes, esophageal temperature lower than 32°C and metabolic acidosis and activated partial thromboplastin time longer than 40s. Such variables can be considered as clinical indicators that would best characterize risk factor “treatment regimen” of the diagnostic classification by NANDA International, Inc. for the clientele studied. In addition, it would support the delimitation of elements for operational definitions related to ECC-induced coagulopathy, considering that the nursing diagnosis risk factor “inherent coagulopathy” does not adequately apply to most cases of excessive bleeding in the postoperative period of cardiac surgery.

The study on the factors of risk for bleeding in cardiac surgery provides relevant information for the validation of the nursing diagnosis and application in the care for people undergoing cardiac surgeries.

## Footnotes

1 Medical Records Departaments
