# Erratum

**DOI:** 10.1590/1518-8345.0000.3147

**Published:** 2019-05-16

Regarding the article “Diagnostic evaluation of risk for bleeding in cardiac surgery with extracorporeal circulation”, with DOI number: 1518-8345.2523.3092, published in Rev. Latino-Am. Enfermagem, 2018;26:e3092, page 5:

Where was written:

**Table 2:** Analysis of the association between the risk factor and bleeding, expressed by the Odds ratio. Rio de Janeiro, RJ, Brazil, 2013-2015

Risk factors	Cases	Controls	Odds ratio and significance		
	N	n	OR*	CI^†^ 95%	***p* value** ^‡^
Preoperative period					
Diagnosed systemic high blood pressure	20	170	0.64	(0.20 to 2.06)	0.4627
Platelet antiaggregation < 5 days	22	62	0.90	(0.35 to 2.29)	0.8353
Prothrombin activation time > 14 seconds	16	38	0.75	(0.28 to 1.98)	0.5650
Activated partial thromboplastin time > 40 seconds	13	38	1.24	(0.50 to 3.07)	0.6343
Hematocrit > 30%^§^	0	10	0.35	(0.02 to 6.24)	0.4788
Platelets < 150,000 cells/mm^3||^	6	18	1.72	(0.53 to 5.53)	0.3633
Body mass index < 26.35kg/m²^¶^	19	98	3.64	(1.31 to 10.15)	0.0134**
Intraoperative period					
ECC^††^ > 90 minutes	46	121	3.57	(1.17 to 10.85)	0.0247**
Surgery > 300 minutes	27	72	1.15	(0.49 to 2.71)	0.7364
Esophageal temperature < 32°C^‡‡^	34	55	2.86	(1.16 to 7.00)	0.0215**
Additional hydric volume in ECC^††^	29	78	1.18	(0.50 to 2.79)	0.6993
Heparin reinforcement in ECC^††^	25	54	2.28	(0.96 to 5.40)	0.0607
Postoperative period					
High blood pressure of difficult control	17	45	0.73	(0.23 to 2.27)	0.5951
Metabolic acidosis	18	58	3.50	(1.35 to 9.05)	0.0098**
Hematocrit < 30%^§^	8	14	2.39	(0.80 to 7.14)	0.1172
Platelets < 150,000 cells/mm^3||^	19	42	1.90	(0.80 to 4.50)	0.1409
Activated partial thromboplastin time > 40 seconds	21	39	2.55	(1.08 to 6.03)	0.0324**
Prothrombin activation time > 14 seconds	44	124	1.57	(0.34 to 7.10)	0.5573

*Odds ratio*;* †95% confidence interval; ‡*p* value; §Percentage; ||Cubic millimeters; ¶Kilos per square meter; **Statistical significance; ††Extracorporeal circulation; ‡‡Degrees Celsius

Now Read:

**Table 2:** Analysis of the association between the risk factor and bleeding, expressed by the Odds ratio. Rio de Janeiro, RJ, Brazil, 2013-2015

Risk factors	Cases	Controls	Odds ratio and significance		
	N	n	OR*	CI^†^ 95%	***p* value** ^‡^
Preoperative period					
Diagnosed systemic high blood pressure	20	170	0.64	(0.20 to 2.06)	0.4627
Platelet antiaggregation < 5 days	13	143	0.40	(0.17 to 0.96)	0.040
Prothrombin activation time > 14 seconds	6	59	0.75	(0.28 to 1.98)	0.5650
Activated partial thromboplastin time > 40 seconds	8	55	1.24	(0.50 to 3.07)	0.6343
Hematocrit > 30%^§^	0	10	0.35	(0.02 to 6.24)	0.4788
Platelets < 150,000 cells/mm^3||^	4	20	1.72	(0.53 to 5.53)	0.3633
Body mass index < 26.35kg/m²^¶^	19	98	3.64	(1.31 to 10.15)	0.0134**
Intraoperative period					
ECC^††^ > 90 minutes	20	112	3.57	(1.17 to 10.85)	0.0247**
Surgery > 300 minutes	13	97	1.15	(0.49 to 2.71)	0.7364
Esophageal temperature < 32°C^‡‡^	16	79	2.86	(1.16 to 7.00)	0.0215**
Additional hydric volume in ECC^††^	14	104	1.18	(0.50 to 2.79)	0.6993
Heparin reinforcement in ECC^††^	14	73	2.28	(0.96 to 5.40)	0.0607
Postoperative period					
High blood pressure of difficult control	4	41	0.73	(0.23 to 2.27)	0.5951
Metabolic acidosis	8	24	3.50	(1.35 to 9.05)	0.0098**
Hematocrit < 30%^§^	5	19	2.39	(0.80 to 7.14)	0.1172
Platelets < 150,000 cells/mm^3||^	11	59	1.90	(0.80 to 4.50)	0.1409
Activated partial thromboplastin time > 40 seconds	12	54	2.55	(1.08 to 6.03)	0.0324**
Prothrombin activation time > 14 seconds	22	168	1.57	(0.34 to 7.10)	0.5573

*Odds ratio*;* †95% confidence interval; ‡*p* value; §Percentage; ||Cubic millimeters; ¶Kilos per square meter; **Statistical significance; ††Extracorporeal circulation; ‡‡Degrees Celsius

